# Phenotype-informed management of preoperative asymptomatic isolated muscular calf vein thrombosis in patients undergoing primary total knee arthroplasty under standardized thromboprophylaxis: a retrospective cohort study

**DOI:** 10.3389/fmed.2026.1813969

**Published:** 2026-04-09

**Authors:** Zhenbao Lu, Qijin Wang, Xu Wang, Yuhua Feng, Xiaohong Fan, Qiujin Xia, Jiliang Chen, Hongkuan Lin, Chengshou Lin, Qingshan Xu, Cuihua Yuan

**Affiliations:** 1 Department of Orthopaedics, Affiliated Mindong Hospital, Fujian Medical University, Ningde, Fujian, China; 2 School of New Energy and Intelligent Manufacturing, Ningde Vocational and Technical College, Ningde, Fujian, China

**Keywords:** duplex ultrasonography, enhanced recovery after surgery, isolated distal deep vein thrombosis, muscular calf vein thrombosis, risk stratification, thromboprophylaxis, thrombus phenotype, total knee arthroplasty

## Abstract

**Background:**

Limited evidence exists regarding the management of preoperative screen-detected (by duplex ultrasonography) asymptomatic isolated muscular calf vein thrombosis (MCVT) before total knee arthroplasty (TKA). We evaluated the outcomes when TKA was performed as scheduled, without preoperative therapeutic-dose anticoagulation, using a standardized thromboprophylaxis and enhanced recovery after surgery (ERAS) pathway.

**Methods:**

Consecutive adults undergoing primary unilateral TKA from 2019 to 2023 received bilateral duplex ultrasonography within 5 days before surgery. The MCVT group comprised patients with asymptomatic isolated gastrocnemius or soleal vein thrombosis, with no involvement of axial deep calf veins or the popliteal vein; the control group had no thrombosis. All patients received a standardized pharmacological prophylaxis regimen (enoxaparin in-hospital followed by rivaroxaban after discharge, total duration 14 days), along with mechanical prophylaxis and early mobilization. The primary outcome was symptomatic venous thromboembolism (VTE) within 90 days. Secondary outcomes included duplex ultrasonography findings on postoperative day 5, bleeding events [defined by International Society on Thrombosis and Hemostasis (ISTH) criteria], Knee Society Score (KSS) at 90 days, and length of stay.

**Results:**

Among 454 patients included, 44 (9.7%) had an isolated preoperative MCVT. No patients in either group developed symptomatic VTE or pulmonary embolism (0/44 vs. 0/410, 95% CI 0.0%–8.0% and 0.0%–0.9%). Routine duplex ultrasonography on postoperative day 5 showed no thrombus progression or new deep vein thrombosis (DVT) in either group. No ISTH-defined major bleeding or clinically relevant non-major bleeding (CRNMB) occurred. KSS outcomes and length of stay were similar between groups (all *P* > 0.05).

**Conclusion:**

Under a protocolized ERAS pathway with combined mechanical prophylaxis and standard chemoprophylaxis, we observed no clinically evident thrombotic or bleeding events within 90 days in patients with asymptomatic isolated preoperative MCVT who proceeded to TKA without delay or preoperative therapeutic-dose anticoagulation. These findings are descriptive and hypothesis-generating and cannot exclude rare but clinically important differences; prospective multicenter studies are needed to quantify rare events and clarify generalizability across thrombus profiles and prophylaxis regimens.

## 1 Introduction

Total knee arthroplasty (TKA) is the definitive treatment for end-stage knee osteoarthritis (OA), providing reliable improvements in pain, function, and quality of life (1). As the population ages and surgical volumes increase, perioperative risk mitigation has become increasingly central to TKA care pathways (2). Despite standardized prophylaxis, venous thromboembolism (VTE) and bleeding remain clinically important concerns. In cohorts using instrumental preoperative duplex screening, deep vein thrombosis (DVT) is detected in approximately 6.7% of patients awaiting knee arthroplasty, and many of these thrombi are clinically silent at the time of detection (3). In contemporary practice, the 30-day incidence of clinically recognized postoperative VTE is around 1.19%, whereas bleeding events occur in about 3.43% of patients (4), underscoring the challenge of preventing thrombosis while avoiding unnecessary bleeding. Against this background, a practical perioperative dilemma is how to manage an incidentally detected, asymptomatic isolated muscular calf vein thrombosis (MCVT) before scheduled TKA.

Several major guidelines, including the International Consensus Meeting on VTE (ICM-VTE) and the UK’s National Institute for Health and Care Excellence (NICE) guidance, recommend individualized VTE risk assessment followed by combined mechanical and pharmacological prophylaxis, along with early postoperative mobilization (5, 6). Routine preoperative whole-leg duplex ultrasonography is not universally recommended and practice varies across institutions. During the study period, our center performed bilateral whole-leg duplex ultrasonography within 5 days before elective TKA as part of routine preoperative assessment to document baseline venous status and to identify occult proximal DVT. If anticoagulation is contraindicated, mechanical prophylaxis is advised as the primary approach (5, 6). Evidence on pharmacological prophylaxis remains mixed: in carefully selected low- to moderate-risk cohorts, aspirin and direct oral anticoagulants (DOACs) have shown comparable effectiveness and safety, whereas the CRISTAL randomized trial reported aspirin to be less effective than enoxaparin for preventing symptomatic VTE within 90 days (7–9). Accordingly, agent selection should align with institutional protocols and patient-level risk. Uncertainty also persists regarding how to manage thrombosis detected before surgery. Most orthopedic and general thromboprophylaxis guidelines classify DVT as proximal or distal based on anatomy, yet none offer a dedicated pathway for MCVT (5, 6). By comparison, the European Society for Vascular Surgery (ESVS) guidelines consider MCVT a distal DVT and propose that anticoagulation decisions be guided by symptoms and high-risk features for thrombus progression (10). Consequently, uncertainty persists when an asymptomatic preoperative MCVT is detected in a TKA candidate.

In patients undergoing TKA, preoperative screen-detected asymptomatic isolated MCVT is not uncommon. These thrombi are typically confined to the gastrocnemius or soleal veins. Detection rates vary across cohorts, likely influenced by patient risk and screening timing/intensity (11–13). In other lower-extremity orthopedic surgery with routine duplex screening, Park et al. reported a 14.7% DVT detection rate, with 92.7% confined to the calf level and most cases asymptomatic (14). Using a phenotype-based anatomical stratification approach, this anatomical confinement defines a distinct ultrasound thrombus phenotype that may carry a different short-term risk profile than thrombosis involving the axial deep calf veins. Compared with thrombosis involving the axial deep calf veins (posterior tibial, peroneal, or tibioperoneal trunk), MCVT has been associated with a lower risk of proximal extension and pulmonary embolism (15, 16). However, this observation is based largely on small retrospective studies, which limits confidence in extrapolating the findings to elective TKA populations. The American College of Chest Physicians (CHEST) guidelines classify MCVT as a distal DVT and recommend serial imaging surveillance over immediate therapeutic anticoagulation for low-risk isolated distal thrombosis (17). However, this recommendation is based primarily on non-surgical patient cohorts, and its applicability to elective TKA is uncertain because perioperative factors can alter both thrombotic and bleeding risk profiles. Variations in chemoprophylaxis regimens (7, 18), tourniquet use (19), and ERAS pathway implementation (20) may further shift the risk balance, leaving a practical evidence gap in phenotype-informed management of preoperative MCVT in the TKA setting.

Therefore, we conducted an exploratory single-center retrospective cohort study to describe the observed short-term thrombotic and bleeding outcomes when scheduled primary unilateral TKA proceeded without preoperative therapeutic-dose anticoagulation under a standardized thromboprophylaxis and ERAS pathway in patients with screen-detected asymptomatic isolated MCVT confined to the gastrocnemius/soleal veins (no axial deep calf vein or popliteal involvement), compared with patients without preoperative thrombosis.

## 2 Materials and methods

### 2.1 Study design and ethical considerations

This single-center retrospective cohort study was conducted at a tertiary hospital. Ethical approval was obtained from the institutional review board (Approval No. K2025102102), and all procedures were conducted in accordance with the Declaration of Helsinki. The study is reported following the Strengthening the Reporting of Observational Studies in Epidemiology (STROBE) guidelines. Written informed consent for use of anonymized clinical data was obtained from all patients upon admission. No animal studies were involved.

### 2.2 Patient selection

From January 2019 to September 2023, we screened all consecutive adult patients at our institution who were undergoing primary unilateral TKA for primary knee OA for eligibility. This period was selected because routine whole-leg duplex ultrasonography within 5 days before surgery and a consistent institutional perioperative pathway (standardized thromboprophylaxis plus ERAS) were implemented throughout these years.

Patients were included if they met all of the following criteria: (1) age ≥ 18 years; (2) diagnosis of primary knee OA; (3) bilateral lower-extremity venous duplex ultrasonography within 5 days before surgery; and (4) complete clinical records with follow-up through 90 days postoperatively.

Patients were excluded if they met any of the following criteria: (1) underwent revision TKA or simultaneous bilateral TKA; (2) had a documented history of VTE; (3) were on chronic oral anticoagulant or antiplatelet therapy; (4) had malignancy, coagulopathy, or active autoimmune disease; (5) were lost to follow-up within 90 days after surgery; or (6) had a preoperative DVT extending into the popliteal or more proximal veins, or a distal DVT involving the axial deep calf veins (posterior tibial, peroneal, or tibioperoneal trunk). These criteria defined a carefully screened, relatively low-risk elective TKA cohort for the present exploratory analysis.

### 2.3 Preoperative assessment and grouping

Preoperative bilateral lower-extremity duplex ultrasonography was routinely performed in all elective TKA candidates within 5 days before surgery as part of an institutional policy during the study period, using a single system (Philips Q5, Philips, Netherlands). Examinations were interpreted in routine clinical practice by an experienced sonographer blinded to the study hypothesis and postoperative outcomes; no independent second reading or formal interobserver/intraobserver reliability assessment was performed. Examinations followed a standardized whole-leg duplex protocol, with compression ultrasonography from the common femoral and femoral veins to the popliteal vein and systematic assessment of the calf venous system, including the posterior tibial, peroneal, tibioperoneal trunk, and intramuscular calf veins (gastrocnemius and soleal veins); color and spectral Doppler were used as needed. MCVT was defined as a non-compressible intraluminal filling defect in a gastrocnemius or soleal vein, and isolated MCVT required absence of thrombus in the axial deep calf veins and the popliteal or more proximal veins. Quantitative thrombus characteristics (e.g., length, multiplicity, clot burden) and acute-versus-chronic features were not routinely documented.

Patients were categorized using a predefined preoperative duplex ultrasonography phenotype framework. The MCVT group comprised patients with screen-detected asymptomatic isolated thrombosis confined to the gastrocnemius or soleal veins, with explicit exclusion of thrombus involvement in the axial deep calf veins (posterior tibial, peroneal, or tibioperoneal trunk) and no extension to the popliteal or more proximal veins; patients had no calf swelling, pain, or tenderness at baseline. The control group included patients with no DVT detected on the same whole-leg preoperative ultrasonography protocol. In patients meeting the asymptomatic isolated MCVT phenotype definition, preoperative therapeutic-dose anticoagulation was not administered, and TKA proceeded as scheduled under the institutional standardized perioperative protocol.

### 2.4 Surgical and perioperative management

All surgeries were performed by the same senior arthroplasty surgeon using a standardized technique. A midline anterior incision with a medial parapatellar approach was employed, and a cemented posterior-stabilized prosthesis was implanted. No tourniquet was used, no surgical drain was placed, and anesthesia consisted of general anesthesia combined with peripheral nerve blocks.

Both groups received the same perioperative VTE prophylaxis regimen. Enoxaparin 4,000 anti-Xa IU was administered subcutaneously once daily from 24 h after surgery until hospital discharge as a fixed prophylactic dose under the institutional protocol. After discharge, rivaroxaban 10 mg once daily was continued to complete a total of 14 days of pharmacological prophylaxis, consistent with commonly adopted arthroplasty prophylaxis recommendations for a minimum 10–14-day course (5, 6). Mechanical prophylaxis measures included graduated compression stockings and intermittent pneumatic compression. Patients began ambulation on postoperative day 1, and multimodal analgesia and rehabilitation were implemented as part of an enhanced recovery after surgery (ERAS) pathway. In-hospital administration was verified from medication records. Post-discharge adherence to rivaroxaban and mechanical prophylaxis was assessed during scheduled follow-up contacts based on patient report.

### 2.5 Data collection and outcome measures

Baseline variables recorded were age, sex, body mass index (BMI), American Society of Anesthesiologists (ASA) class, operative side, comorbidities (hypertension, diabetes mellitus, and lower-extremity varicose veins), and key preoperative laboratory values (D-dimer and hemoglobin). The primary outcome was symptomatic VTE within 90 days after surgery, defined as any symptomatic DVT and/or pulmonary embolism.

Both groups underwent routine duplex ultrasonography surveillance on postoperative day 5, and additional imaging was obtained if new symptoms suggested a thromboembolic event. Thrombus progression was defined as any extension into the popliteal vein or more proximal veins, or a newly detected DVT on follow-up ultrasound. Bleeding events were classified according to the International Society on Thrombosis and Hemostasis (ISTH) criteria as either major bleeding or clinically relevant non-major bleeding (CRNMB). Early functional recovery was assessed with the Knee Society Score (KSS) preoperatively and again at 90 days postoperatively.

Follow-up data were collected via outpatient clinic visits and telephone interviews; patients lost to follow-up within 90 days were excluded. During follow-up, patients were asked about any interim care at other institutions. Electronic medical records were reviewed to capture events within our hospital system during the 90-day postoperative period.

### 2.6 Statistical Analysis

All analyses were performed using SPSS version 26.0 (IBM Corp., Armonk, NY, United States). Data distribution was evaluated with the Shapiro–Wilk test. Continuous variables with a normal distribution are presented as mean ± standard deviation (SD) and were compared using an independent-samples *t*-test. Continuous variables with a non-normal distribution are presented as median (interquartile range, IQR) and were compared using a Mann–Whitney U test. Categorical variables are shown as counts (percentages) and were compared using a chi-square test or Fisher’s exact test, as appropriate.

Given the retrospective observational design and the anticipated rarity of symptomatic thrombotic and major bleeding events under a protocolized arthroplasty pathway, no *a priori* target sample size was specified. All consecutive eligible cases within the predefined study period were included. Event analyses were primarily descriptive. For outcomes with zero events in one or both groups, relative measures (risk ratio/odds ratio) and regression modeling were not performed. We reported the absolute risk difference with a two-sided 95% confidence interval, and calculated exact two-sided 95% confidence intervals for event rates using the Clopper–Pearson method to quantify the range compatible with the observed data.

## 3 Results

### 3.1 Baseline characteristics

**FIGURE 1:**
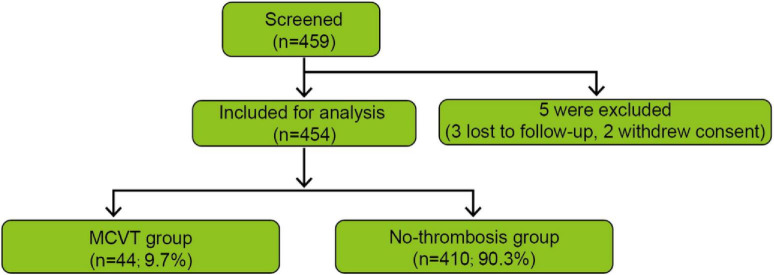
Flow diagram of patient screening, exclusions, and cohort allocation.

As shown in Figure 1, of 459 patients screened, 5 were excluded (three lost to follow-up and two withdrew consent), leaving 454 for analysis (44 MCVT and 410 no-thrombosis); all included patients completed 90-day follow-up.

Baseline characteristics were similar between the MCVT and no-thrombosis groups (Table 1). The median age was 69 years in both groups. There were no significant differences between groups in sex distribution, BMI, ASA class, operative side, comorbidities (hypertension, diabetes mellitus, and varicose veins), or key preoperative laboratory values (D-dimer and hemoglobin) (all *P* > 0.05).

**TABLE 1:** Baseline demographic and preoperative clinical characteristics.

Variable	MCVT group	No-thrombosis group	*P*-value
Age (years)	69.0 [65.0, 73.0]	69.0 [65.0, 73.0]	0.848^c^
Sex			
Female, *n* (%)	29 (65.9%)	262 (63.9%)	0.792^b^
Male, *n* (%)	15 (34.1%)	148 (36.1%)	
BMI (kg/m^2^)	25.1 ± 3.8	25.8 ± 3.7	0.230^a^
Hypertension, *n* (%)	30 (68.2%)	265 (64.6%)	0.639^b^
Diabetes mellitus, *n* (%)	8 (18.2%)	102 (24.9%)	0.325^b^
Varicose veins, *n* (%)	12 (27.3%)	98 (23.9%)	0.620^b^
ASA class			
I–II, *n* (%)	40 (90.9%)	368 (89.8%)	0.982^b^
III–IV, *n* (%)	4 (9.1%)	42 (10.2%)	
Preoperative D-dimer (mg/L FEU)	0.5 [0.3, 0.7]	0.5 [0.3, 0.7]	0.301^c^
Preoperative hemoglobin (g/L)	131.6 ± 13.9	130.0 ± 13.4	0.437^a^
Operative side			
Left, *n* (%)	23 (52.3%)	206 (50.2%)	0.798^b^
Right, *n* (%)	21 (47.7%)	204 (49.8%)	

^a^*P*-values were calculated using the independent-samples *t*-test.

^b^Chi-square test.

^c^Mann–Whitney U test. MCVT, muscular calf vein thrombosis; BMI, body mass index; ASA, American Society of Anesthesiologists; Hb, hemoglobin; FEU, fibrinogen equivalent units.

### 3.2 Primary outcome: symptomatic VTE within 90 days

No symptomatic VTE was observed within 90 days in either group (0/44 vs. 0/410). The exact two-sided 95% confidence interval for the event rate was 0.0%–8.0% in the MCVT group and 0.0%–0.9% in no-thrombosis group. The absolute risk difference (MCVT minus control) was 0.0% (95% CI −0.9% to 8.0%). Despite the event-free observation, the upper bound in the MCVT group remains clinically non-trivial, and the study is underpowered to exclude clinically meaningful differences.

### 3.3 Secondary outcomes

#### 3.3.1 Thrombus progression on postoperative day 5 (duplex ultrasonography)

**FIGURE 2:**
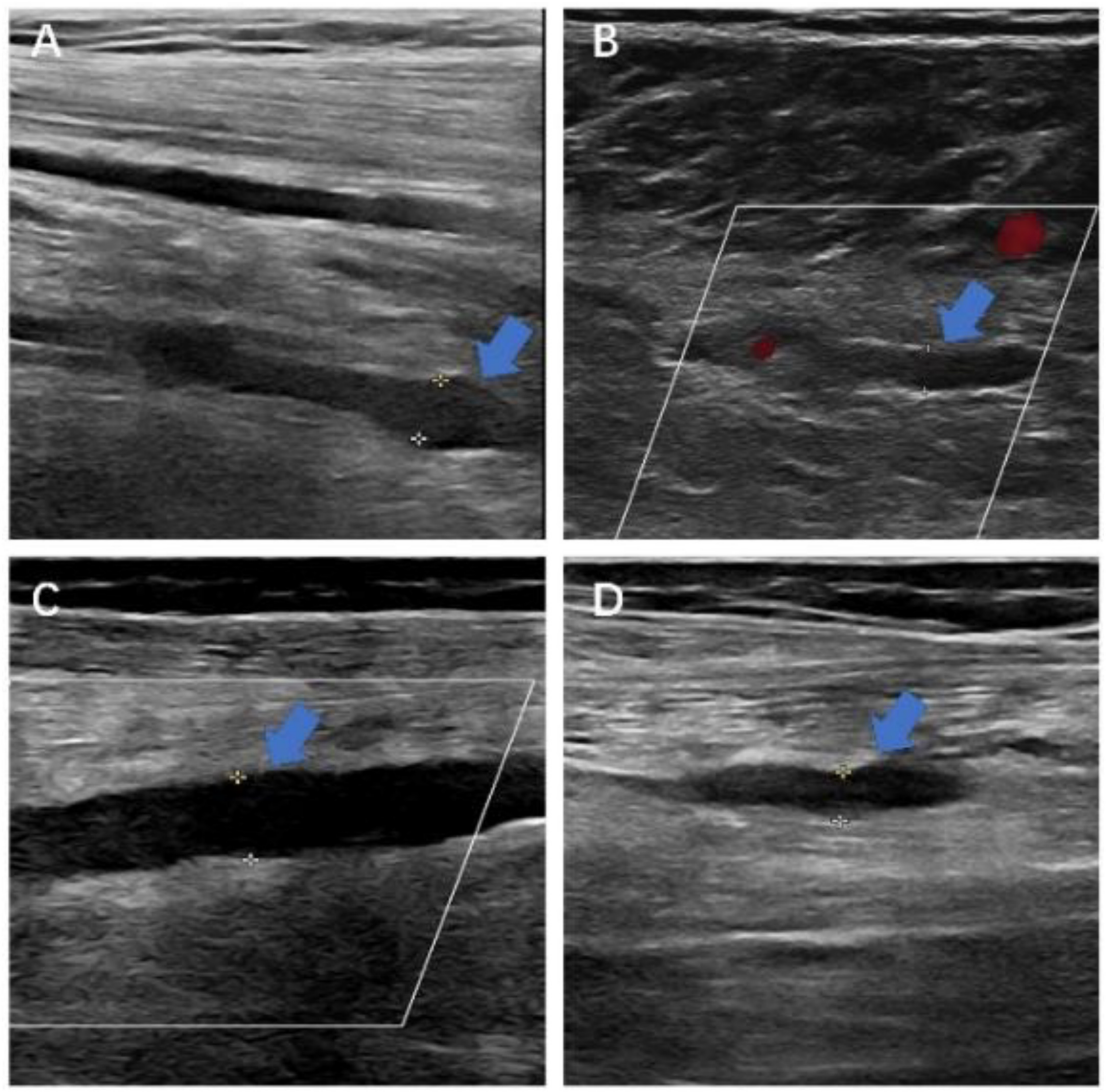
Representative preoperative duplex ultrasonography images of isolated muscular calf vein thrombosis (MCVT) detected before total knee arthroplasty (TKA). **(A–D)** Duplex scans demonstrate a non-compressible gastrocnemius or soleal vein with an intraluminal low-echogenic filling defect (arrow), without involvement of the popliteal or more proximal veins.

Routine whole-leg duplex ultrasonography on postoperative day 5 showed no thrombus progression, proximal propagation, or newly detected postoperative DVT in either group (MCVT: 0/44; no-thrombosis: 0/410). In the MCVT group, residual intramuscular thrombus without extension was present in 30 patients (68.2%), whereas complete sonographic resolution was observed in 14 (31.8%). Figure 2 presents representative preoperative duplex images of isolated MCVT.

#### 3.3.2 Bleeding events

No ISTH-defined major/CRNMB occurred within 90 days, and no transfusion or reoperation for hemostasis was required (MCVT 0/44 vs. no-thrombosis 0/410). Minor wound events were self-limited: oozing 7/44 (15.9%) vs. 44/410 (10.7%) and small ecchymoses 15/44 (34.1%) vs. 131/410 (32.0%).

#### 3.3.3 Functional recovery at 90 days (KSS)

The KSS improved significantly from baseline to 90 days in both groups (within-group comparison, *P* < 0.001 for each). The median increase in KSS was similar between the two groups: ΔKSS was 36.0 (32.8–45.0) in the MCVT group versus 39.0 (34.0–44.0) in the no-thrombosis group (*P* = 0.488). The absolute KSS at 90 days was also similar between groups (83.3 ± 6.9 vs. 83.4 ± 5.9, *P* = 0.936; Table 2).

**TABLE 2:** Clinical outcomes at 90 days after total knee arthroplasty (TKA).

Variable	MCVT group	No-thrombosis group	*P*-value
Preoperative KSS	45.0 (40.8–51.0)	44.0 (41.0–49.0)	0.312^b^
KSS at 90 days	83.3 ± 6.9	83.4 ± 5.9	0.936^a^
ΔKSS (90-day minus preoperative)	36.0 (32.8–45.0)	39.0 (34.0–44.0)	0.488^b^

^a^*P*-values were calculated using the independent-samples *t*-test.

*^b^*Mann–Whitney U test. KSS, Knee Society Score; MCVT, muscular calf vein thrombosis.

#### 3.3.4 Length of hospital stay

Length of hospital stay was comparable between the groups (median 7 [6–8] days for both; *P* = 0.786). The mean length of stay was 6.64 ± 1.14 days in the MCVT group and 6.58 ± 1.16 days in the no-thrombosis group. No prolonged hospitalization due to thrombus-related complications was observed within 90 days postoperatively.

## 4 Discussion

To address the lack of TKA-specific guidance for preoperative screen-detected MCVT, we conducted a single-center retrospective cohort study of 454 primary TKA cases managed under a standardized protocol incorporating combined pharmacological and mechanical thromboprophylaxis within an ERAS pathway. Forty-four patients with screen-detected isolated asymptomatic MCVT underwent TKA as scheduled without preoperative therapeutic-dose anticoagulation. No symptomatic VTE was observed within 90 days in either group, and routine postoperative day-5 duplex ultrasonography demonstrated no thrombus progression or proximal propagation. Bleeding outcomes, 90-day KSS improvement, and length of stay were comparable between groups. Within this protocolized pathway, both groups were event-free; these findings are descriptive and hypothesis-generating. The study was not designed for equivalence/non-inferiority, and clinically meaningful differences cannot be excluded. Our evaluation primarily reflects clinically evident outcomes rather than comprehensive thrombus dynamics.

From a phenotype-based anatomical stratification perspective, our study represents an initial step based on anatomical ultrasound phenotyping rather than a comprehensive multivariable precision risk-stratification model. Preoperative DVT has been reported in approximately 2.6%–6.7% of patients scheduled for TKA (3). However, evidence specific to MCVT remains limited, and in several cohorts intramuscular calf vein thrombosis accounts for a substantial proportion of distal DVTs detected on screening (11). This variability has contributed to uncertainty regarding the optimal timing of surgery when a distal thrombosis is identified preoperatively. Some studies support proceeding with surgery without delay if thromboprophylaxis is protocol-driven, whereas others advocate deferring surgery to first administer therapeutic anticoagulation (21, 22). These divergent recommendations likely reflect heterogeneity in whether proximal thrombi (e.g., popliteal DVT) are included, how “distal” DVT is defined (particularly whether axial deep calf veins are included), and differences in prophylaxis intensity and perioperative protocols (23). In this context, our study provides TKA-specific data on isolated asymptomatic MCVT managed without preoperative therapeutic anticoagulation under combined mechanical and pharmacological prophylaxis with early mobilization. Prior work suggests that MCVT is less prone to proximal extension than axial deep calf vein thrombosis and that these calf thrombi often undergo organization, recanalization, or resorption over the short- to mid-term, although arthroplasty-specific data remain sparse (24, 25). Taken together, these observations suggest that a risk-balanced strategy may be considered, rather than automatically escalating anticoagulation for all patients with isolated MCVT.

Clinically, this study addresses a practical dilemma in arthroplasty: when an isolated asymptomatic MCVT is detected on preoperative screening ultrasound, is it necessary to delay surgery for therapeutic anticoagulation, or can TKA proceed as scheduled under standard prophylaxis? In our cohort, no symptomatic VTE within 90 days, no day-5 thrombus progression on duplex ultrasonography, and no ISTH-defined major bleeding or CRNMB were observed when TKA proceeded as scheduled without preoperative therapeutic-dose anticoagulation. This event-free observation does not establish equivalence. These findings are most applicable to carefully screened elective TKA candidates with isolated gastrocnemius/soleal MCVT managed within a standardized ERAS and chemoprophylaxis pathway, and should not be extrapolated to higher-risk patients or axial/proximal DVT phenotypes. Because institutional practices vary (for example, some centers use aspirin-only prophylaxis or do not perform routine postoperative ultrasound), extrapolation to less intensive pathways should be undertaken with caution. Surgical timing decisions should remain individualized, especially when higher-risk features are present.

A pragmatic proposal for phenotype-based anatomical stratification: To facilitate translation into routine practice, a pragmatic approach to preoperative calf vein thrombosis in elective TKA may prioritize (i) confirming thrombus phenotype (muscular-vein only vs. axial deep calf vein involvement and any extension to the popliteal or more proximal veins), (ii) screening for higher-risk features (symptoms, suspected hypercoagulable states, active malignancy, or inability to mobilize early), and (iii) aligning prophylaxis intensity with the individual bleeding risk and institutional ERAS resources. In patients meeting the predefined low-burden muscular-vein phenotype definition and able to follow an ERAS pathway with standardized chemoprophylaxis and early mobilization, proceeding with surgery on schedule may be considered, whereas escalation of anticoagulation and/or surgical delay may be reserved for higher-risk phenotypes.

Several biologically plausible mechanisms could be hypothesized to explain the observed short-term stability of the muscular-vein phenotype; however, these remain speculative and were not directly tested in this study. First, MCVT often results from local venous stasis and valvular dysfunction in the intramuscular calf veins, and its hemodynamic characteristics may confer a lower tendency for proximal propagation than thromboses in the axial deep calf veins (direct mechanistic evidence in arthroplasty patients is limited) (15, 24). In addition, local inflammatory and fibrinolytic activity may promote thrombus organization, recanalization, and resorption over time (25). In our perioperative programme, the combination of pharmacological and mechanical prophylaxis with early postoperative mobilization (from day 1) could plausibly reduce venous stasis; whether this influences thrombus dynamics in muscular veins requires dedicated study. Conversely, postponing surgery solely due to a screen-detected MCVT may have practical trade-offs, including disruption of planned perioperative pathways. However, we did not include a delayed-surgery comparator, and whether postponement alters thrombotic risk in elective TKA remains unknown. Evidence linking surgical delay to higher VTE/DVT incidence mainly comes from fracture/trauma populations and may not generalize to elective arthroplasty settings (26, 27). These considerations underscore the need to balance thrombosis and bleeding risks within a structured perioperative framework.

Several limitations should be acknowledged. This retrospective single-center cohort is subject to residual confounding, and the MCVT subgroup was relatively small (*n* = 44), limiting power to detect very rare thrombotic or bleeding events. Follow-up was limited to 90 days, and although whole-leg duplex ultrasonography was routinely performed on postoperative day 5, subsequent imaging was symptom-driven, so late asymptomatic distal thrombosis may have been missed. Moreover, because universal preoperative duplex screening was an institutional policy in this cohort, the cost-effectiveness and potential false-positive implications were not evaluated and external validity to centers without routine screening may be limited. Post-discharge adherence was assessed by patient report and was not objectively verified. The eligibility criteria also yielded a carefully screened low-risk cohort, and the findings should not be extrapolated to higher-risk patients or to axial/proximal DVT phenotypes. Events occurring outside our institution may have been missed. In addition, thrombus phenotyping was restricted to anatomical location, as quantitative ultrasound features (e.g., thrombus length, multiplicity, and echogenicity-based chronicity) were not systematically captured, constraining more granular risk stratification; formal interobserver/intraobserver reliability testing for duplex interpretation was not performed. Laterality, muscular-vein subtype distribution, and clot-burden changes confined to muscular veins were not available for analysis. Future multicentre prospective studies with harmonized phenotype definitions and prespecified imaging schedules are warranted to quantify rare events, assess the influence of prophylaxis intensity and ERAS implementation, and support translation into a pragmatic phenotype-informed perioperative management pathway, ideally incorporating standardized ultrasound feature sets, clinical risk markers, causal-inference methods, and value-based outcomes (including readmissions and healthcare costs).

In summary, under a standardized combined thromboprophylaxis regimen and ERAS pathway, we observed no symptomatic VTE within 90 days, no day-5 thrombus progression on duplex ultrasonography, and no ISTH-defined major bleeding or CRNMB in patients with asymptomatic isolated preoperative MCVT; 90-day functional recovery was similar between groups. Given the event-free results and limited sample size, these findings are hypothesis-generating and cannot exclude rare but clinically important complications; thus, this phenotype may be considered only in carefully selected patients managed within comparable protocol-driven settings. However, multicentre prospective studies with predefined stratification by thrombus location and burden are warranted to confirm the risk of rare complications, evaluate longer-term outcomes, and refine perioperative management for different thrombus profiles.

## 5 Conclusion

Within a standardized perioperative pathway incorporating combined thromboprophylaxis and ERAS, we observed no 90-day symptomatic thrombotic events and no ISTH-defined major/CRNMB in patients with asymptomatic isolated MCVT confined to the gastrocnemius/soleal veins, and early functional recovery was similar to that of patients without thrombosis. These descriptive, hypothesis-generating data are limited to short-term (90-day) outcomes, do not demonstrate equivalence, and cannot exclude rare but clinically important differences; multicentre prospective studies with prespecified stratification and endpoints are needed to quantify rare events and clarify longer-term outcomes across thrombus profiles.

## Data Availability

The raw data supporting the conclusions of this article will be made available by the authors, without undue reservation.
